# Round the Hospitals

**Published:** 1921-02-19

**Authors:** 


### ROUND THE HOSPITALS.

The death of Sister Edith Johncock, R.R.C., Matron of the British Hospital in Nazareth, is a very serious loss to the mission field in which she laboured so long. She began her long career of service as a slum officer under the Salvation Army. After nine years of this work she entered the Royal Victoria Hospital at Dover, and on receiving her certificate of training accepted work at Nazareth under the Edinburgh Medical Missionary Society sixteen years ago. Here she remained till her death from influenza after a short illness on December 7. The organisation of the hospital from its first inception, and its gradual expansion, owed much to her talents. The training of the native staff was one of her great achievements. Hers was a name to conjure with throughout Galilee, where in every village there were men, women, and children who had come under her care. Her most noble years, perhaps, were those spent during the war in Nazareth, far from friends, under Turkish rule, when single-handed she spent herself for the wounded, and ministered faithfully to friend and foe alike.

Amongst the published list of grants from the United Services Fund, of which the chairman is General Lord Byng, is one of £15,000 towards the formation of a club for nurses out of the £200,000 which has been allocated for immediate expenditure by that Fund for the benefit of ex-Service women, and one of £10,000 for a holiday home for nurses. The name of the Club is not mentioned, but we have grounds for stating that it is the United Nursing Services Club to which this grant has been made. In connection with the holiday home, we learn that delightful premises have been bought at Folkestone. We hope the United Nursing Services Club may now be able to go rapidly ahead. Will it be a race between it and the Cowdray Club as to which shall first be ready to open its doors?

The Visiting Committee of the Highgate Hill Infirmary has made its report following on full and exhaustive inquiry into the whole of the matters raised by Miss L. M. Wamsley, an inspector of the Ministry of Health, to which we referred in our issue of January 29 (p. 415). Certain state-

**ROUND THE HOSPITALS—(*continued*).**

ments in her report caused a feeling of indignation among the nursing staff, which, it is hoped, the report of the Visiting Committee, which was adopted by the Guardians at their meeting on the 11th instant, will allay. The report, while stating concisely that, after the fullest investigation, the Committee cannot find any justification for the statements complained of—i.e., that many of the nurses think of little else but off-duty time and money, of which there is no lack, and that they come on duty tired and jaded after late hours and constant excitement—deals in detail with the whole of Miss Wamsley's report. The Committee states there appears to have been a misunderstanding over some of the items which could have been explained by the principal officers, while other points are due to the transitory stage then existing at the infirmary. This transitory stage refers, no doubt, to the 48-hour working week which is at present being tried at Islington Infirmary. The present experimental arrangement has not proved quite satisfactory, as it interferes with the continuity of observation of a patient by the nurse, and the Committee is considering the best method of rearranging the hours of duty. The inquiry has brought to light certain matters which require adjustment, which have already been taken in hand. The best friends of the infirmary will therefore not regret the inquiry, which, so far as the Committee is concerned, vindicates the nursing staff, whilst its report admits that Miss Wamsley's report contains "otherwise valuable recommendations." We understand that the report and findings of the Visiting Committee have been sent to the Ministry of Health.

---

It is good news that new regulations under the Public Health Act for the control of Nursing and Maternity Homes will come into force throughout the South African Union on April 1, 1921. In such matters the authorities of the Overseas Dominions take action, while on this side we are still deliberating and foreseeing obstacles to reform. The measure is so far a tentative one. It provides for registration and inspection, but does not provide for the issue of licences. A register of all nursing and maternity homes will be kept at Pretoria, and all who apply for registration must submit to inspection at any time by a medical officer of the Health Department or by a whole-time M.O.H. in the service of a local authority. All proprietors of homes are required to furnish annual returns giving minute particulars of cases treated in the home. Special precautions are being taken to ensure a return of all still-births, it being suspected that certain homes in the larger towns are being used for purposes of abortion. We have constantly urged the need in London and other cities for a measure of this description. The courageous manner in which the South African authorities have tackled the matter ought to stimulate the London County Council to use their powers in the same direction.

The Leeds Trained Nurses' Institute is one of the few private nursing societies in possession of a pension fund. At the annual meeting recently announcement was made that the pension fund has been converted into a trust fund, the investments of which cannot be used for any other purpose than the payment of pensions to nurses. During the year 464 cases were attended, while 346 had to be declined for lack of nurses. This would seem to indicate the need for enlarging the staff. There is a much appreciated district nursing branch, under which 3,126 cases were attended.

---

The Salford Guardians are faced with the necessity for making considerable additions to the Hope Union Infirmary. A new laundry is needed, and will cost £15,000. Extensions to the nurses' home to admit more probationers are estimated to cost £70,000, and other alterations will bring the total cost to nearly £100,000. It is not unnatural in present circumstances that opposition to the scheme has made itself felt. One member of the Board argued that arrangements might be made for the nurses to live outside the institution, and quoted the approval of the Medical Superintendent in favour of his contention that the living-in system that has prevailed up to now will soon be a thing of the past. Apart from other considerations, it would probably cost more in the end to give every nurse an extra £2 a week for board and lodging than to enlarge the home, and this the Chairman wisely pointed out. But if suitable accommodation can be had outside as a temporary nurses' home so much the better.

---

The London County Council has entered upon a very thorny path in enforcing its new system of licensing and inspecting massage and kindred establishments. Already a group of very shady establishments have expired as an effect of the regulations now in force. The Council has power to refuse to grant or renew a licence under certain conditions, but the aggrieved person may appeal to a metropolitan police magistrate and subsequently to quarter sessions. A recent case in which the magistrate declined to uphold the decision of the L.C.C. authorities has opened up the difficulties of effective inspection, and it seems as though more stringent regulations as to the management and medical superintendence of these establishments may be necessary to secure freedom from abuses without the kind of inspection so strongly objected to by English lawyers.

---

A somewhat unusual legacy has been bequeathed to the Stamford, Rutland and General Infirmary by one of its trustees, Mr. Ernest William Proby Conant, J.P., who by his will left £150 for the endowment of an annual examination, with prizes for proficiency in the science and practice of hypodermic injection, to be open to all nurses regularly employed at the infirmary. Mr. Conant in addition bequeathed £200 to the infirmary for general purposes.

